# Disruption of Thyroid Hormone Receptor–Mediated Transcription and Thyroid Hormone–Induced Purkinje Cell Dendrite Arborization by Polybrominated Diphenyl Ethers

**DOI:** 10.1289/ehp.1002065

**Published:** 2010-09-22

**Authors:** Kingsley Ibhazehiebo, Toshiharu Iwasaki, Junko Kimura-Kuroda, Wataru Miyazaki, Noriaki Shimokawa, Noriyuki Koibuchi

**Affiliations:** 1 Department of Integrative Physiology, Gunma University Graduate School of Medicine, Maebashi, Japan; 2 Department of Developmental Morphology, Tokyo Metropolitan Institute for Neuroscience, Tokyo, Japan; 3 Laboratory of Environmental Health Sciences, Center for Disease Biology and Integrative Medicine, Graduate School of Medicine, University of Tokyo, Tokyo, Japan

**Keywords:** flame retardants, gene regulation, neurodevelopment, neurogenesis, polybrominated diphenyl ethers (PBDEs), thyroid

## Abstract

**Background:**

Polybrominated diphenyl ethers (PBDEs) have been used as flame retardants and are becoming a ubiquitous environmental contaminant. Adverse effects in the developing brain are of great health concern.

**Objective:**

We investigated the effect of PBDEs/hydroxylated PBDEs (OH-PBDEs) on thyroid hormone (TH) receptor (TR)-mediated transcription and on TH-induced dendrite arborization of cerebellar Purkinje cells.

**Methods:**

We examined the effect of PBDEs/OH-PBDEs on TR action using a transient transfection-based reporter gene assay. TR–cofactor binding was studied by the mammalian two-hybrid assay, and TR–DNA [TH response element (TRE)] binding was examined by the liquid chemiluminescent DNA pull-down assay. Chimeric receptors generated from TR and glucocorticoid receptor (GR) were used to identify the functional domain of TR responsible for PBDE action. The change in dendrite arborization of the Purkinje cell in primary culture of newborn rat cerebellum was also examined.

**Results:**

Several PBDE congeners suppressed TR-mediated transcription. The magnitude of suppression correlated with that of TR–TRE dissociation. PBDEs suppressed transcription of chimeric receptors containing the TR DNA binding domain (TR-DBD). We observed no such suppression with chimeras containing GR-DBD. In the cerebellar culture, PBDE significantly suppressed TH-induced Purkinje cell dendrite arborization.

**Conclusions:**

Several PBDE congeners may disrupt the TH system by partial dissociation of TR from TRE acting through TR-DBD and, consequently, may disrupt normal brain development.

Polybrominated diphenyl ethers (PBDEs) have been used as flame retardants in a wide range of consumer and household goods (Hale et al. 2003). Their chemical stability allows PBDEs to be global environmental contaminants (Costa and Giordano 2007; Tseng et al. 2008). An alarming increase in PBDE concentrations in humans has been reported (Hites 2004; Law et al. 2003). Among congeners, 2,2′,4,4′-tetra-BDE (BDE47), 2,2′,4,4′,5-penta-BDE (BDE99), 2,2′,4,4′,6-penta-BDE (BDE100), 2,2′,4,4′,5,5′-hexa-BDE (BDE153), 2,2′,4,4′,5,6-hexa-BDE (BDE154), and 2,2′,3,3′,4,4′,5,5′,6,6′-deca-BDE (BDE209) are predominant in human tissues (Costa et al. 2008; Hites 2004). PBDEs and their hydroxylated metabolites (OH-PBDEs) have been detected in fetal blood and liver, and in maternal breast milk and placenta (Daniels et al. 2010; Gómara et al. 2007; Schecter et al. 2003). Furthermore, PBDEs can cross the blood–brain barrier to accumulate in the central nervous system (Naert et al. 2007). On the whole, the body burden of PBDEs is increasing (Hites 2004; Law et al. 2003; Sjödin et al. 2004).

PBDEs/OH-PBDEs have been implicated in developmental neurotoxicity. Neonatal exposure induces neurobehavioral changes in rodents (Branchi et al. 2003; Eriksson et al. 2001; Gee and Moser 2008). Such exposure also reduces long-term potentiation and postsynaptic protein levels in the hippocampus (Dingemans et al. 2007) and affects intracellular calcium homeostasis and induces oxidative stress (Kodavanti and Ward 2005). However, mechanisms of PBDE action in developing brain are not yet fully understood.

PBDEs/OH-PBDEs are structurally similar to the thyroid hormones (THs) 3,5,3′,5′-tetraiodo-l-thyronine [thyroxine (T_4_)] and 3,5,3′-triiodo-l-thyronine [triiodothyronine (T_3_)] (Schriks et al. 2007). Thus, PBDEs may act through the TH system (Birnbaum and Staskal 2004). TH is critical for normal brain development (Porterfield 2000), and TH deficiency during the perinatal critical period causes abnormal brain development known as cretinism in humans (Koibuchi and Chin 2000; Rabié et al. 1977). Decreased serum levels of T_4_ have been reported after perinatal exposure to various PBDEs in rodents (Costa and Giordano 2007; Rice et al. 2007; Zhou et al. 2001, 2002). In addition, OH-PBDEs have been shown to bind to the human TH receptor (TR) (Kitamura et al. 2008; Kojima et al. 2009; Li et al. 2010). A recent reporter gene assay study showed that BDE206 inhibits TR-mediated transcription (Schriks et al. 2007). These results suggest that PBDEs/OH-PBDEs may affect TH-regulated signal transduction pathways at multiple levels. However, mechanisms of PBDE/OH-PBDE action on TR-mediated transcription have not yet been fully clarified.

To study the mechanisms of TH action in the developing brain, we have used rodent cerebellum as a model system. Because perinatal hypothyroidism is associated with decreased dendrite arborization and synaptogenesis of Purkinje cells (Nicholson and Altman 1972a, 1972b), Purkinje cells are considered a good model to examine various TH actions in developing brain. Using this system, Kimura-Kuroda et al. (2007) observed the modification of TH-induced dendrite arborization by hydroxylated polychlorinated biphenyls (OH-PCBs).

The present study was designed to examine the mechanisms of PBDE action on modulation of TR-mediated gene expression and on morphological alterations in cultured Purkinje cells. Because the structure of PBDE is similar to that of PCB, we hypothesized that PBDE could suppress TR-mediated TH action.

## Materials and Methods

### Chemicals

We purchased T_3_, T_4_, and dexamethasone (Dex) from Sigma Chemical Co. (St. Louis, MO, USA). All PBDE/OH-PBDE congeners were purchased from AccuStandard Chemicals (New Haven, CT, USA). Purity was > 99% for all PBDE congeners and > 98% for all OH-PBDEs. DE-71 contains BDE99 (44%), BDE47 (32%), BDE100 (9%), BDE153 (4%), and other PBDEs (total 11%). Ethanol (Wako, Osaka, Japan) was used as a vehicle. All compounds were completely dissolved in ethanol at concentrations < 10^−4^ M and stored at −20°C. Dilutions were made from stock solutions immediately before use, and repeated freezing and thawing were avoided. PBDE congeners used in the present study were carefully selected to reflect their abundance in the environment and in humans (Hites 2004; Law et al. 2003).

### Plasmids

Expression vectors for TRβ1, TRα1, and glucocorticoid receptor (GR) were described previously (Iwasaki et al. 2001, Koibuchi et al. 1999). The luciferase (LUC) reporter constructs containing chick lysozyme–TH response element (TRE) thymidine kinase (TK)-LUC (F2-TRE), artificial direct repeat TRE–DR4-TK-LUC (DR4-TRE), and 2× palindrome (Pal)-TK-LUC (Pal-TRE) in the PT109 vector were described by Koibuchi et al. (1999). A 5× upstream activating sequence (UAS)-TK-LUC reporter plasmid in the PT109 vector and a mouse mammary tumor virus (MMTV) promoter containing glucocorticoid response element (GRE), which is fused to the LUC promoter (MMTV-LUC), were described by Iwasaki et al. (2001). Expression vectors for Gal4–DNA binding domain (DBD)-fused steroid receptor coactivator-1–nuclear binding domain-1 (SRC-1–NBD-1; aa 595–780) (Takeshita et al. 2002) and VP16-TRβ1–ligand binding domain (LBD) (Miyazaki et al. 2004) were described previously. The Gal4 blank, Gal4-SMRT (silencing mediator for retinoid and thyroid hormone receptors; aa 1669–2507), and Gal4–N-CoR (nuclear receptor corepressor; aa 1579–2454) were described previously by Takeshita et al. (2002). The glutathione *S*-transferase (GST)-fused TRβ1 was described by Qiu CH et al. (2009). Chimeric receptors were generated from TR and GR as described by Miyazaki et al. (2008).

### Clonal cell culture

CV-1 monkey fibroblast-derived cells were maintained in Dulbecco’s modified Eagle’s medium supplemented with 10% fetal bovine serum deprived of small lipophilic hormone by treating with resin and activated charcoal, at 37°C and 5% CO_2_.

### Transient transfection assay

CV-1 cells were plated in 24-well plates 2 days before transfection using the calcium phosphate coprecipitation method (Miyazaki et al. 2008). The total amount of DNA per well was balanced by adding pcDNA3 plasmids (Invitrogen, San Diego, CA, USA). Cytomegalovirus β-galactosidase plasmid was used as an internal control. Sixteen to 24 hr after transfection, wells were refilled with fresh medium containing the indicated concentrations of ligand and/or PBDEs and incubated for 24 hr. For TH treatment, we used T_3_ because it is bioactive and because CV-1 cells do not contain deiodinase activity. Cells were then harvested to measure the LUC activities as described elsewhere (Miyazaki et al. 2008). The LUC activities were normalized to β-galactosidase activity and calculated as relative LUC activities. All transfection experiments were repeated at least three times in triplicate, and data represent mean ± SE of one experiment. The method of mammalian two-hybrid assay has been described previously (Baniahmad et al. 1995; Carey et al. 1989).

### Liquid chemiluminescent DNA pull-down assay

(LCDPA). The LCDPA was used to measure nuclear receptor–DNA binding in solution (Iwasaki et al. 2008). Briefly, a GST-fused TR (GST-TR) bound to glutathione-Sepharose beads was incubated with a digoxigenin (DIG)-labeled double-stranded DNA fragment containing a TRE, in protein–DNA binding buffer. After extensive washing, protein–DNA binding on beads was detected using anti-DIG antibody conjugated to alkaline phosphatase, which was then measured by a chemiluminescent reaction using a luminometer. The LCDPA was performed at least three times, and data represent the mean ± SE of one experiment.

### Primary culture

The animal experimentation protocol was approved by the Animal Care and Experimentation Committee, Gunma University. Pregnant Wistar rats were purchased from Japan SLC, Inc. (Hamamatsu, Japan). Animals were treated humanely and with regard for alleviation of suffering. Newborn rats were decapitated under diethyl ether anesthesia on postnatal day 1. We followed the culture protocol described by Kimura-Kuroda et al. (2007). Briefly, cerebella were digested with 0.2 U/mL papain (Worthington, Lakewood, NJ, USA). Dissociated cells were then suspended in a serum-free medium without THs, plated at a density of 2.0 × 10^5^ cells/0.2 mL in poly-l-lysine–coated wells of chamber slides (8-mm-diameter wells, NUNC Lab-Tek; Nalge Nunc International, Rochester, NY, USA), and cultured overnight in a 5% CO_2_ incubator. Then, T_4_ and/or PBDEs were added to the culture medium. Half of the medium was replaced with fresh medium every 3–4 days, and cells were cultured for 17 days. The pH of the culture medium was not altered after PBDE treatment. For TH treatment, T_4_ was used in primary culture because, physiologically, it is preferentially transported to brain through the blood–brain barrier, and primary culture contains the whole subset of cerebellar cells, including astrocytes. Physiologically, T_4_ is taken up by astrocytes and deiodinated to T_3_, which is then transferred to neurons.

### Immunocytochemistry for calbindin to analyze Purkinje cell development

Immunocytochemistry of the cultured cells was described previously by Kimura-Kuroda et al. (2007). Briefly, we used a mouse monoclonal anti-calbindin-28K antibody (1:1,000; McAB 300; Swant, Bellinzona, Switzerland) and fluorescein isothiocyanate (FITC)-labeled donkey anti-mouse antibody (1:200; Molecular Probes, Eugene, OR, USA). The morphological changes were examined under a laser confocal scanning microscope (FV1000D spectral-type inverted IX81 microscope; Olympus, Tokyo, Japan). To quantify dendrite arborization, we manually traced the outline of the cell and dendritic branches of 10 randomly selected Purkinje cells from each experiment, and the area was computed using NIH Image software (Vischer 2006). Data shown represent mean ± SE of one experiment. We performed more than three independent experiments and confirmed consistency of the results.

### Statistical analysis

We analyzed treatment effects using analysis of variance (ANOVA). Post hoc comparison was made using Bonferroni’s test, and *p*-values < 0.05 are considered significant.

## Results

### Congener-specific suppression of TR-mediated transcription by PBDEs/OH-PBDEs

**Figure 1:**
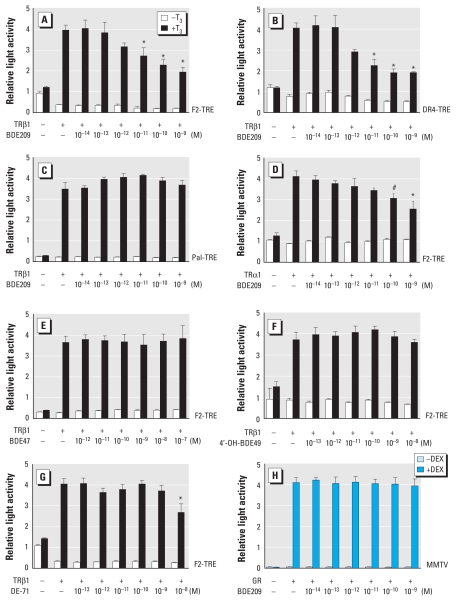
Congener-specific suppression of TR-mediated transcription by PBDEs/OH-PBDEs. Expression plasmids encoding TRβ1 (*A*–*C*, *E*–*G*) or TRα1 (*D*; 10 ng) were cotransfected with F2-TRE (*A*, *D*–*G*), DR4-TRE (*B*), or Pal-TRE (*C*; 100 ng) into CV-1 cells. Cells were incubated with or without 10^−7^ M T_3_ and indicated amounts of PBDEs/OH-PBDEs. (*H*) Expression plasmids encoding GR (10 ng) were cotransfected with MMTV (GRE)-LUC reporter plasmids (100 ng) into CV-1 cells. Cells were cultured in the absence or presence of Dex (10^−7^ M) and indicated concentrations of BDE209. Total amounts of DNA for each well were balanced by adding vector pcDNA3. Data are mean ± SE of experiments performed in triplicate. *p < 0.01, and ^#^*p* < 0.05 by ANOVA, compared with TRβ1 (+), T_3_ (+), and BDE209 (−) in *A*, *B*, and *D* and DE-71 (−) in *G*.

**Figure 2:**
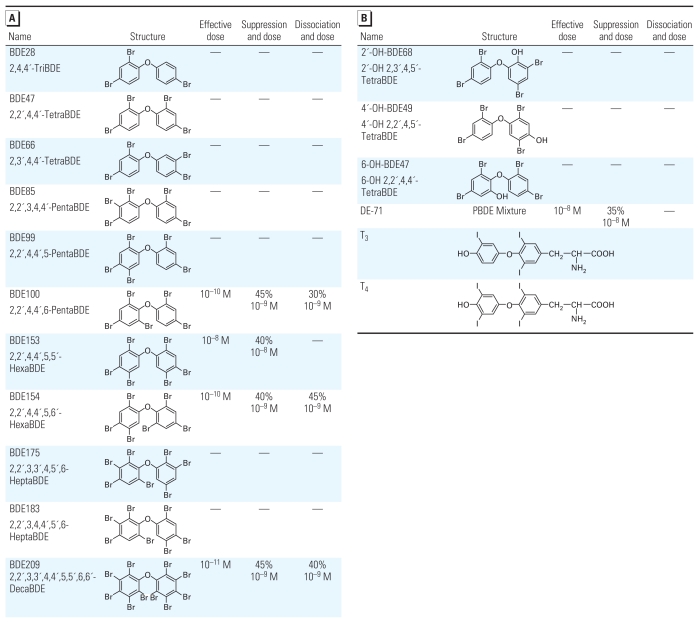
Effects of PBDE congeners (*A*) or OH-PBDEs and a PBDE mixture (*B*) on TR-mediated transcription and TR–TRE binding. Transient transfection-based reporter gene assays (Figure 1A) and LCDPA (Figure 3E) were carried out using each chemical. Results of minimum effective dose, maximum suppression (%) and dose, and maximum dissociation (%) and dose are listed. —, no effect.

We examined the effect of various PBDE congeners, OH-PBDE metabolites, and PBDE mixtures on TR-mediated transcription in fibroblast-derived CV-1 cells using the transient transfection-based reporter gene assay. Representative results are shown in Figure 1 [see also Supplemental Material, Figures 3 and 4 (doi:10.1289/ehp.1002065)]. The tested compounds are listed in Figure 2. Among the PBDE compounds used, we observed the greatest magnitude of suppression with BDE209 and BDE100 (45% at 10^−9^ M; Figures 1A and 2A). BDE209 had the lowest effective dose (10^−11^ M) of the compounds evaluated (Figures 1A and 2). BDE209 suppressed TRβ1-mediated transcription on F2-TRE and DR4-TRE but not on Pal-TRE (Figure 1A–C). PBDEs/OH-PBDEs did not alter transcription levels when TH was absent (Figure 1). BDE209 also suppressed TRα1-mediated transcription (Figure 1D), but the magnitude of suppression was less than that for TRβ1-mediated transcription (Figure 1A). Several congeners (e.g., BDE47) did not suppress TR action (Figure 1E). We also examined the effect of hydroxylated metabolites, because several OH-PCBs suppressed TR action with a greater magnitude than did their parent PCBs (Miyazaki et al. 2004). However, as shown in Figures 1F and 2B [see also Supplemental Material, Figure 4D,E (doi:10.1289/ehp.1002065)], no OH-PBDEs suppressed TR action. DE-71, a commercial PBDE mixture, suppressed TR-mediated transcription to some extent (Figures 1G and 2B), although BDE100 and BDE153 make up < 15% of the mixture. Trypan blue exclusion confirmed that the PBDEs/OH-PBDEs did not induce cell death under the concentrations used in this study (data not shown). Also, we examined the effect of PBDEs/OH-PBDEs on reporter vector expression without TR transfection and observed no significant change (data not shown). On the other hand, BDE209 did not suppress GR-mediated transcription (Figure 1H), suggesting that the suppressive effects may be TR specific.

### PBDEs did not alter ligand-dependent cofactor recruitment to TRβ1 in CV-1 cells

**Figure 3:**
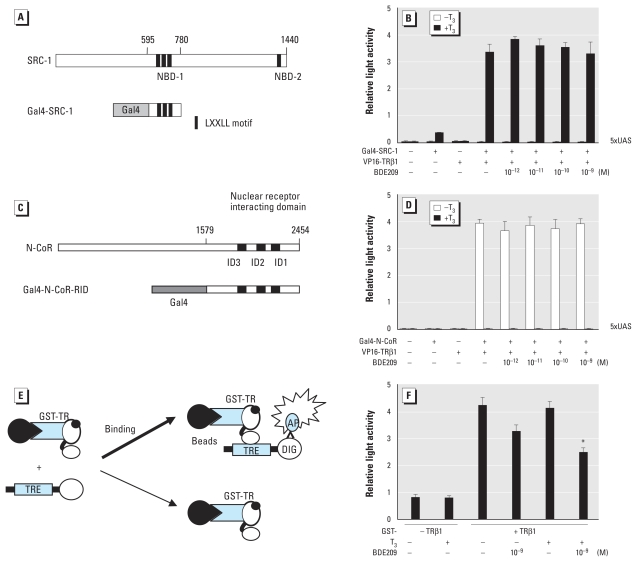
The effect of PBDE on TR cofactor or TR–TRE binding. (*A*) Schematic diagram of Gal4–SRC-1–NBD-1. Functionally active LXXLL motifs (Leu-Xaa-Xaa-Leu-Leu) are located in central (residues 633–637, 690–694, and 749–753) and C-terminal (residues 1434–1438) regions termed NBD-1 and NBD-2, respectively. Gal4–SRC-1–NBD-1 contains amino acid residues 595–780 of SRC-1. (*B*) BDE209 did not affect SRC-1 binding to liganded TR in CV-1 cells. Expression plasmids encoding Gal4–DBD–fused SRC-1–NBD-1 (10 ng) were cotransfected with VP16 constructs (50 ng) and 5× UAS-TK-LUC reporter plasmids (170 ng) into CV-1 cells. Cells were incubated with or without T_3_ (10^−7^ M) and indicated concentrations of BDE209. (*C*) Schematic diagram of Gal4–N-CoR, which contains amino acid residues 1579–2454 of N-CoR. (*D*) BDE209 did not recruit N-CoR to TR in CV-1 cells. Expression plasmids harboring Gal4-DBD-fused N-CoR (100 ng) were cotransfected with VP16-TRβ1-LBD (50 ng) and 5× UAS-TK-LUC (100 ng) into CV-1 cells. Cells were incubated with or without T_3_ (10^−7^ M) and indicated concentrations of BDE209. (*E*) Schematic diagram showing the LCDPA method. GST-TRβ1 bound to Sepharose beads was incubated with DIG-labeled F2-TRE in protein–DNA binding buffer with or without T_3_ (10^−6^ M) and indicated concentrations of PBDE. (*F*) The effect of BDE209 on TRβ1–TRE binding using LCDPA. In *B, D*, and *F*, data represent mean ± SE of experiments performed in triplicate. *p < 0.01 by ANOVA, compared with GST-TRβ1 (+), T_3_ (+), and PBDE (−).

We investigated the effect of PBDEs on binding between TR and coactivator using the mammalian two-hybrid assay with Gal4–SRC-1– NBD-1 and VP16-TRβ1-LBD (Figure 3A). Transcriptional activation by Gal4–SRC-1–NBD-1 and VP16-TRβ1-LBD with T_3_ was not affected by BDE209 or any other compounds used in the present study (Figure 3B), indicating that these compounds do not dissociate TR–coactivator binding.

Another possibility of PBDE action may be recruitment of corepressor complexes to liganded TR. Thus, we performed a mammalian two-hybrid assay using Gal4–N-CoR (Figure 3C) or Gal4-SMRT [see Supplemental Material, Figure 1A (doi:10.1289/ehp.1002065)], and VP16-TRβ1. Without T_3_, Gal4–N-CoR and VP16-TRβ1 activated transcription (Figure 3D), whereas we observed no activation with T_3_. Activation was not altered by BDE209 or any other compounds used in the present study (Figure 3D). PBDEs also did not recruit SMRT without T_3_ (see Supplemental Material, Figure 1B). These results indicate that PBDEs do not recruit corepressors to TR.

### PBDEs partially dissociate TR from TRE

To examine whether PBDEs dissociate TR from TRE *in vitro*, we performed LCDPA (Figure 3E). BDE209 (10^−9^ M) partially dissociated TR from TRE (40%) in the presence of T_3_ (10^−6^ M; Figure 3F). Together with mammalian two-hybrid studies, these results indicate that the suppression of TR-mediated transcription by BDE209 was due to partial dissociation of TR from TRE. On the other hand, BDE47, which did not suppress TR-mediated transcription, did not dissociate TR from TRE [see Supplemental Material, Figure 2 (doi:10.1289/ehp.1002065)]. However, BDE153, which suppresses TR-mediated transcription with a higher dose (10^−8^ M) than other compounds, did not dissociate TR–TRE binding, although BDE153 did not alter TR–cofactor binding either (data not shown). The results of LCDPA are summarized in Figure 2.

### TR-DBD may be responsible for the effect of PBDEs on TR action

**Figure 4:**
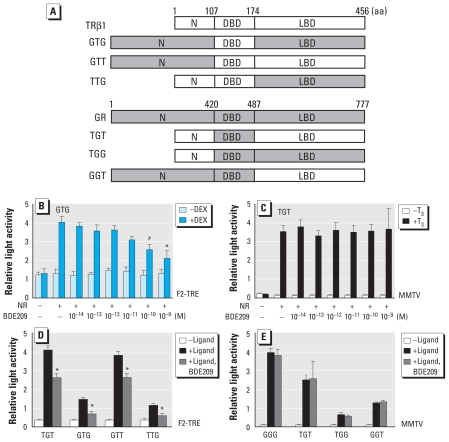
TR-mediated transcription is altered by PBDE through TR-DBD. (*A*) Schematic diagram of chimeric protein used in the present study. Abbreviations: G, GR; T, TR; N, N-terminal domain. (*B,C*) Representative examples of PBDE actions on chimeric receptor-induced transcription (*B*, GTG; *C*, TGT). (*D*,*E*) Effects of BDE209 on transcription through chimeric receptors containing TR-DBD (*D*) or GR-DBD (*E*). For *B–E*, chimeric receptors (10 ng) were cotransfected with F2-TRE (*B,D*) or MMTV (GRE)-LUC (*C,E*) reporter plasmid (100 ng) into CV-1 cells and incubated with or without Dex (10^−7^ M) or T_3_ (10^−7^ M; *C*), and 10^−14^ to 10^−9^ M BDE209. Data represent mean ± SE of experiments performed in triplicate. *p < 0.01, and ^#^*p* < 0.05 by ANOVA, compared with GTG (+), Dex (+), and BDE209 (−) in *B*, and compared with chimeric receptors (+), ligand (+), and BDE209 (−) in *D*.

Next, we attempted to identify the functional domains of TR responsible for PBDE action using chimeric receptors (Figure 4A). Although all chimeric receptors harboring TR-DBD show suppression of transcription by BDE209 regardless of the difference in LBD (Figure 4B,D), we observed no such suppression with receptors harboring DBD of GR (Figure 4C,E), indicating that the site of PBDE action may be DBD of TR.

### PBDEs disrupt TH-dependent dendrite arborization of cerebellar Purkinje cells

**Figure 5:**
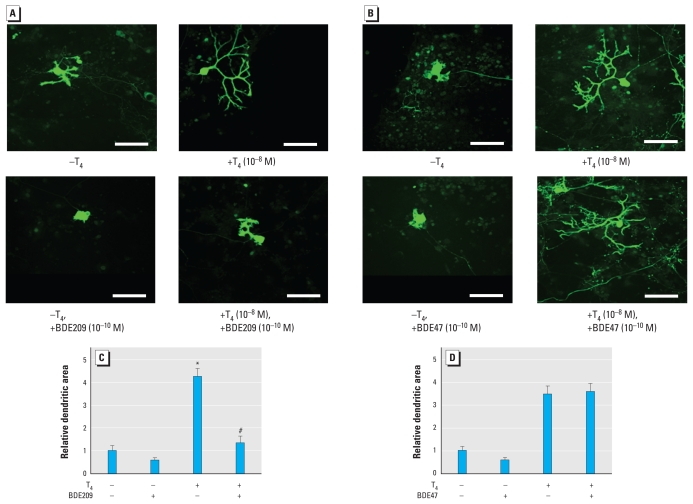
Effect of PBDEs on dendrite arborization of Purkinje cell (17 days in culture). (*A*,*B*) Photomicrographs showing the effect of PBDEs on Purkinje cell morphology. BDE209 (10^−10^ M; *A*) or BDE47 (10^−10^ M; *B*) was added to the culture in the absence or presence of T_4_ (10^−8^ M), and immunocytochemistry was performed using anti-calbindin antibody to visualize Purkinje cells. Bars = 50 μm. (*C,D*) Change in dendritic areas of Purkinje cells by BDE209 (*C*)or BDE47 (*D*). In *C* and *D*, data are expressed as mean ± SE (*n* = 10 determinations) from one experiment and represent at least three independent experiments. *p < 0.01 by ANOVA, for T_4_ (+)/BDE209 (−) compared with T_4_ (−)/BDE209 (−). ^#^*p* < 0.01 by ANOVA, for T_4_ (+)/BDE209 (+) compared with T_4_ (+)/BDE209 (−).

We studied the effect of several PBDE/OH-PBDE compounds on T_4_-induced dendrite arborization of cerebellar Purkinje cells in primary culture. Seventeen days after the onset of culture, cells were fixed and immunostained with anti-calbindin antibody. Addition of BDE209 (10^−10^ M) to the culture together with T_4_ inhibited development of Purkinje cell dendrites. The dendrites showed very poor growth, and the secondary branches were especially small (Figure 5A); the area of these Purkinje cell dendrites was significantly reduced (Figure 5A,C). In the absence of T_4_, BDE209-treated Purkinje cells showed almost complete absence of dendrites (Figure 5A). On the other hand, addition of BDE47 (10^−10^ M), which does not suppress TR action, did not inhibit Purkinje cell dendrite development (Figure 5B). The dendritic area was not different from that without BDE47 (Figure 5D). These data indicate that the PBDE congener that suppresses TR-mediated transcription also inhibits TH-mediated Purkinje cell dendrite arborization.

### Increasing the concentration of T_4_ could not fully rescue the suppression of T_4_-mediated Purkinje cell dendrite arborization by PBDEs

**Figure 6:**
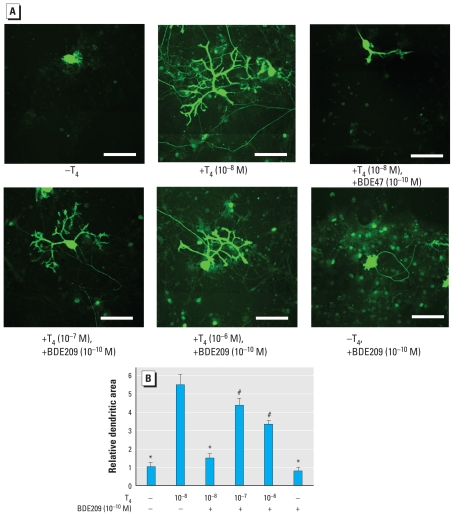
Increasing the concentration of T_4_ partially reverses the suppressive effects of BDE209 on Purkinje cell dendrite arborization (17 days in culture). (*A*) Photomicrographs showing the effect of BDE209 and T_4_ on Purkinje cell morphology. BDE209 (10^−10^ M) was added to the culture in the absence or presence of T_4_ (10^−8^ M to 10^−6^ M), and immunocytochemistry was performed using anti-calbindin antibody to visualize Purkinje cells. Bars = 50 μm. (*B*) Effect of BDE209 and T_4_ on dendritic area of Purkinje cells. Data are mean ± SE (*n* = 10 determinations) from one experiment and represent at least three independent experiments. *p < 0.01, and ^#^*p* < 0.05 by ANOVA, compared with T_4_ (10^−8^ M)/BDE209 (−).

When we increased the T_4_ concentration to investigate the change in PBDE-mediated suppression of Purkinje cell dendrite arborization, increasing amounts of T_4_ significantly ameliorated the effect of 10^−10^ M BDE209. However, even at 10^−6^ M, T_4_ could not fully rescue the suppression (Figure 6). Purkinje cells had fewer secondary branches and bifurcations and less dendrite area than those cultured with 10^−8^ M T_4_ alone (Figure 6), indicating that T_4_ can only partially rescue BDE209 action.

## Discussion

In the present study, PBDE induced congener-specific suppression of TR-mediated transcription. Of the congeners evaluated, BDE209 (deca-BDE) and BDE100 showed the greatest suppression (45% at 10^−9^ M), and BDE209 had the lowest effective dose (10^−11^ M). Such effects may be partially induced by dissociation of TR from TRE. The site of PBDE action may be on TR-DBD. Furthermore, PBDE disrupted Purkinje cell dendrite arborization.

Because of structural similarity, we initially hypothesized that the suppression of TR-mediated transcription by PBDEs could be due to competitive inhibition of T_3_ binding to TR or alteration of TR–cofactor binding. However, the mechanism of action is completely different from our initial hypothesis. Our experiments, involving the mammalian two-hybrid assay, transient transfection assays using chimeric receptors, and LCDPA, clearly demonstrated that the mechanism of action of PBDEs is similar to that of PCBs that we previously reported (Miyazaki et al. 2008). However, the BDE153 congener did not cause dissociation of the binding by LCDPA, although it significantly suppressed TR action. It should be noted that BDE153 did not alter TR–cofactor binding (data not shown). Because a greater amount of BDE153 (10^−8^ M) is required to induce suppression in CV-1 cells, its effect may be too weak to detect by *in vitro* assay.

In the chimeric receptor experiments, all chimeric receptors containing TR-DBD showed suppression of transcription by PBDE regardless of the difference in N- or C-terminus (Figure 4B,D), whereas no such suppression was observed with receptors harboring GR-DBD (Figure 4C,E), indicating that the site of BDE209 action may be TR-DBD. These findings are similar to those of our previous studies showing that PCBs may disrupt TR action by partial dissociation of TR–TRE binding, acting via TR-DBD (Miyazaki et al. 2008). Thus, PBDEs and PCBs could affect TR-mediated gene expression via a common pathway. PBDEs and PCBs have also been shown to affect signal transduction pathways, particularly calcium homeostasis and protein kinase C activity (Kodavanti and Ward 2005; Madia et al. 2004). In contrast, although hydroxylated PCB metabolites have been shown to effectively suppress TR-mediated gene expression (Miyazaki et al. 2008), the present study clearly revealed that only parent PBDE congeners effectively suppressed TR-mediated transcription. Thus, the site of PBDE action in the TR-DBD molecule could be different from that of PCBs because of differences in molecular structure and hydrophilicity. None of the OH-PBDE congeners used in our study significantly suppressed TR-mediated transcription (Figure 2B), although previous reports have suggested that other OH-PBDE congeners, whose concentration in humans is much lower, bound to TR (Kitamura et al. 2008; Qiu X et al. 2009). Recently, Li et al. (2010) showed binding between TRβ1 and some OH-PBDE compounds used in our study. However, they failed to show transcriptional activation or suppression with such compounds. Similar tendencies have been reported, showing that some OH-PBDE compounds—at low doses used in the present study—bind to TR without altering transcriptional activities (Kitamura et al. 2008; Kojima et al. 2009).

Although TH markedly promoted Purkinje cell dendrite arborization, BDE209 inhibited TH-induced dendrite development of Purkinje cells (Figure 5A,C). The effective dose, 10^−10^ M, is similar to that observed in our reporter gene assay. TRs are ubiquitously expressed in most cerebellar cells, including Purkinje cells, during development (Bradley et al. 1992), and previous studies have shown that TH induces Purkinje cell dendrite development via TR (Bradley et al. 1992; Strait et al. 1991). Thus, the inhibitory effects of PBDEs observed in our study could be due to their action on TR-mediated gene expression in Purkinje cells and may consequently disrupt normal brain development. However, increased concentrations of TH could not fully reverse the suppressive effects of BDE209 on TH-mediated Purkinje cell dendrite arborization (Figure 6). Purkinje cells cultured in the presence of 10^−10^ M BDE209 and 10^−7^ M or 10^−6^ M T_4_ showed less arborized dendrites than did those cultured in the presence of 10^−8^ M T_4_ alone (Figure 6), indicating the existence of additional pathways, such as disruption of intracellular signaling pathways that rely on Ca^2+^ homeostasis (Dingemans et al. 2010; Kodavanti and Ward 2005; Londoño et al. 2010; Madia et al. 2004) or induction of cytochrome P450 activities (Zhou et al. 2002). Further studies are required to investigate the synergistic effect of PBDEs on multiple signal transduction pathways. Nevertheless, the present study has provided an important clue to clarify the mechanisms of PBDE action in developing brain.

Because TH also tightly regulates fundamental gene expression both directly and indirectly, not only in the cerebellum but also in other brain regions (Koibuchi et al. 1999, 2001), the inhibitory effects of PBDEs on TR-mediated gene expression may widely disrupt normal brain development via TH-dependent gene regulations. Because PBDEs can be transferred to the developing brain through placenta or breast milk, continuous investigation is absolutely required for further clarification of PBDE action in other brain regions.

## Conclusions

Our study shows that several PBDE congeners suppress TR-mediated gene expression by partial dissociation of TR from TRE through the DBD. At least partly through such a mechanism, PBDEs may inhibit TH-dependent dendrite arborization of cerebellar Purkinje cells. We hope that our study will provide significant clarifications on the mechanisms of PBDE action in the developing brain.
